# 4-Hydroxy-2-Nonenal Promotes Cardiomyocyte Necroptosis *via* Stabilizing Receptor-Interacting Serine/Threonine-Protein Kinase 1

**DOI:** 10.3389/fcell.2021.721795

**Published:** 2021-10-01

**Authors:** Xiaoxuan Zhai, Wenjun Wang, Shukun Sun, Yu Han, Jiaxin Li, Shengchuan Cao, Ruochuan Li, Tonghui Xu, Qiuhuan Yuan, Jiali Wang, Shujian Wei, Yuguo Chen

**Affiliations:** ^1^Department of Emergency and Chest Pain Center, Qilu Hospital, Cheeloo College of Medicine, Shandong University, Jinan, China; ^2^Clinical Research Center for Emergency and Critical Care Medicine, Qilu Hospital, Institute of Emergency and Critical Care Medicine, Cheeloo College of Medicine, Shandong University, Jinan, China; ^3^Key Laboratory of Emergency and Critical Care Medicine, Qilu Hospital, Cheeloo College of Medicine, Shandong University, Jinan, China; ^4^Key Laboratory of Cardiopulmonary-Cerebral Resuscitation Research, Qilu Hospital, Cheeloo College of Medicine, Shandong University, Jinan, China; ^5^The Key Laboratory of Cardiovascular Remodeling and Function Research, Chinese Ministry of Education, Chinese Ministry of Health and Chinese Academy of Medical Sciences, Qilu Hospital, Cheeloo College of Medicine, Shandong University, Jinan, China; ^6^The State and Shandong Province Joint Key Laboratory of Translational Cardiovascular Medicine, Qilu Hospital, Cheeloo College of Medicine, Shandong University, Jinan, China; ^7^Department of Critical Care Medicine, Shandong Provincial Hospital Affiliated to Shandong First Medical University, Jinan, China

**Keywords:** 4-hydroxynonenal (4-HNE), myocardial ischemia – reperfusion injury (MIRI), necroptosis, RIP1 (RIPK1), ubiquitination

## Abstract

**Background:** Necroptosis is a vital regulator of myocardial ischemia/reperfusion (MI/R) injury. Meanwhile, 4-hydroxy-2-nonenal (4-HNE) is abundantly increased during MI/R injury. However, whether 4-HNE induces cardiomyocyte necroptosis during MI/R remains unknown.

**Methods:** To observe the relationship between 4-HNE and necroptosis during MI/R, C57BL/6 mice and aldehyde dehydrogenase 2-transgenic (ALDH2-Tg) mice were both exposed to left anterior descending artery ligation surgery to establish MI/R injury models. For further study, isolated mouse hearts and H9c2 cells were both treated with 4-HNE to elucidate the underlying mechanisms.

**Results:** Necroptosis and 4-HNE were both upregulated in I/R-injured hearts. Cardiomyocyte necroptosis was significantly decreased in I/R-injured hearts from ALDH2-Tg mice as compared with that of wild-type mice. *In vitro* studies showed that necroptosis was enhanced by 4-HNE perfusion in a time- and concentration-dependent manner. Knockdown of receptor-interacting serine/threonine-protein kinase 1 (RIP1) using small interfering RNA (siRNA) prevented 4-HNE-induced cardiomyocyte necroptosis, manifesting that RIP1 played a key role in the upregulation of cell necroptosis by 4-HNE. Further studies found that 4-HNE reduced the protein degradation of RIP1 by preventing K48-polyubiquitination of RIP1.

**Conclusion:** 4-HNE contributes to cardiomyocyte necroptosis by regulating ubiquitin-mediated proteasome degradation of RIP1.

## Introduction

Reperfusion therapy is a medical treatment that restores the coronary artery blood perfusion and is regarded as the most effective intervention for the treatment of myocardial infarction (Yellon and Hausenloy, 2007). However, reperfusion can cause up to 50% at most of the total myocardial injury and results in malignant arrhythmia, myocardial stunning, and impaired heart function (Heusch and Gersh, 2017). Programmed cardiomyocyte death plays an initiating and central role in myocardial ischemia/reperfusion (MI/R) injury but may receive less clinical attention because of its insidious effects on cardiac structure and function (Zhu and Sun, 2018).

Necrosis used to be considered an “unprogrammed” cardiovascular cell death, while apoptosis and autophagy are denoted as “programmed cell death” and has gained a lot of attention. However, recent studies have shown that necroptosis is the most well-defined regulated necrosis that accounts for up to 30% of cell death during MI/R injury (Smith et al., 2007). It is characterized as loss of plasma membrane integrity and decreasing of cellular adenosine triphosphate (ATP) (Linkermann and Green, 2014; Zhang et al., 2018). Necroptosis is initiated by the activation of receptor-interacting serine/threonine-protein kinase 1 (RIP1) that binds to receptor-interacting serine/threonine-protein kinase 3 (RIP3) to form necrosome, followed by activation of downstream mixed lineage kinase domain-like pseudokinase (MLKL) and Ca^2+^/calmodulin-dependent protein kinase II (CaMKII) (Cho et al., 2009; Chen et al., 2014). Thus, RIP1 plays a key role in transferring death signals and determines cell fate (Ofengeim and Yuan, 2013; Orozco et al., 2014).

4-Hydroxy-2-nonenal (4-HNE), one of the most toxic aldehydes, is the major secondary product of lipid peroxidation (Ayala et al., 2014). 4-HNE, at 0.3–5 μM under normal condition, plays physiological roles as a signaling molecule while 4-HNE is increased by 10–100 times under oxidative stress and plays cytotoxic roles by inhibiting gene expression or modifying proteins (Esterbauer et al., 1991; Ayala et al., 2014). During MI/R injury, 4-HNE is increased by 6-fold in reperfusion-injured hearts to 100 μM (Lucas and Szweda, 1999). Accumulation of 4-HNE increases the formation of 4-HNE adducts by binding to various proteins in the heart tissue and promotes cardiac dysfunction mainly through damaging mitochondria, impairing ATP production, and inducing cardiomyocyte death (Hwang et al., 2020; Santin et al., 2020). The role of 4-HNE in cardiomyocyte apoptosis was previously investigated, but the effects of 4-HNE on cardiomyocyte necroptosis remain unknown (Sun et al., 2014).

In this study, the effects of 4-HNE on necroptosis were investigated *in vivo* and *in vitro*. Using multiple approaches, we found that 4-HNE promoted necroptosis during MI/R by combination with RIP1 and then inhibiting the ubiquitination of RIP1.

## Materials and Methods

### Reagents and Antibodies

Receptor-interacting serine/threonine-protein kinase 1 antibody (3493; 1:1,000), RIP3 antibody (15828; 1:1,000), phosphor-RIP1 antibody (p-RIP1; 1:1,000), Ser166 (31122; 1:1,000), CaMKII antibody (3362; 1:1,000), K48-ubiquitin antibody (8081; 1:1,000), and secondary antibodies (1:10,000) were bought from Cell Signaling Technology (Danvers, MA, United States). Phosphor-RIP3 antibody (p-RIP3) (ab195117; 1:1,000), 4-HNE antibody (ab46545; 1:1,000), phosphor-MLKL antibody (p-MLKL) (ab196436; 1:1,000), phosphor-CaMKII antibody (p-CaMKII) (ab32678; 1:1,000) were purchased from Abcam (Cambridge, MA, United States). β-Actin antibody (6008-1-Ig; 1:5,000) and MLKL antibody (66675; 1:1,000) were bought from Proteintech (Rosemont, IL, United States). Cycloheximide (CHX, 25 μg/ml) (5087390001) was bought from Sigma-Aldrich (Steinheim, Germany).

### Animal Myocardial Ischemia/Reperfusion Injury Models

The present study was approved by the Animal Ethics Committee of Qilu Hospital of Shandong University. All procedures were in adherence with the National Institutes of Health guidelines. Aldehyde dehydrogenase 2-transgenic (ALDH2-Tg) mice were from Professor Jun Ren (University of Wyoming). Wild-type (WT) and ALDH2-Tg male mice (8–12 weeks) were used to perform MI/R injury surgery. After being anesthetized with isoflurane inhalation, the hearts were exposed by thoracotomy. The left anterior descending (LAD) artery was tied with 6-0 silk suture for 30 min and loosened for 4 or 24 h. Then, the hearts and blood of mice were harvested for the following experiments.

### Langendorff Model

The Langendorff model was used to examine the direct effects of 4-HNE on the isolated mouse hearts. After being anesthetized and heparinized, the mouse hearts were harvested quickly and installed on the Langendorff apparatus. The isolated hearts were perfused with Krebs–Henseleit buffer (KH buffer) and stabilized at 37°C with 95% O_2_ and 5% CO_2_ for 20 min and then perfused the heart with KH buffer containing 60 μM 4-HNE or vehicles for 1 h. During that period, left ventricular developed pressure (LVDP) and left ventricular pressure rising rate (dp/dt) were recorded and compared with baseline to measure the left ventricular function.

### Infarct Size Measurement and Echocardiography

To determine myocardial infarct size, the LAD artery was tied at previous place and then perfused with 1% Evans blue dye (EBD) through the aorta. After that, the hearts were frozen and cut into 2-mm slices. Then, the slices were incubated in 1% 2,3,5-triphenyl tetrazolium chloride (TTC) solution for 20 min at 37°C incubator. The red and white areas stood for the areas at risk, and the white area stood for the infarction area. After photographing with a camera, the images were analyzed by ImageJ software (NIH).

Cardiac function after MI/R injury including left ventricular fraction shortening (LVFS) and left ventricular ejection fraction (LVEF) was measured by the echocardiographic method (VEVO 2100, VisualSonics, Toronto, ON, Canada).

### Cell Culture

H9c2 cells (ATCC) were cultured in Dulbecco’s modified Eagle’s medium (DMEM) (Gibco, Thermo Fisher Scientific, MA, United States) plus 10% fetal bovine serum and antibiotics. The cells were grown in the humified incubator with 20% O_2_/5% CO_2_ at 37°C. For hypoxia/reoxygenation (H/R) stimulation, the cells were maintained in the hypoxia chamber (2% O_2_, 5% CO_2_) with low glucose and serum-free DMEM overnight and then were moved to normoxic incubator with regular culture medium for different hours (1, 2, 4, or 6 h).

For 4-HNE stimulation, the cells were cultured in regular culture medium with vehicle or diverse concentration of 4-HNE (20, 40, 60, or 80 μM) and kept in incubator for various hours (1, 2, 4, or 6 h).

### Necroptosis Assay

Myocardium necroptosis was measured by EBD-Caveolin 3 (CaV3) double staining. Mice were intraperitoneally given EBD (10 mg/ml) 14 h before the MI/R surgery. After surgerical operation, excised hearts were put into optimal cutting temperature (OCT) compound and were sliced into 5-μm cryosections. Finally, sections were incubated with CaV3 antibody and were imaged using a fluorescence microscope (Olympus, Tokyo, Japan).

Propidium iodide (PI)/Annexin V staining was used to distinguish the necroptotic cells. V-ZAD-FMK (Selleck Chemicals, TX, United States) was added to inhibit caspases accompanied with vehicle or 4-HNE, then the cells were harvested and washed twice. Cells were resuspended at the concentration of 1 × 10^6^/ml and then were incubated with PI and Annexin V at 37°C for 20 min. The cells were detected using flow cytometry and were analyzed with CytExpert software (Beckman Coulter, IN, United States).

### Receptor-Interacting Serine/Threonine-Protein Kinase 1 Knockdown

To silence the *RIP1* gene, the H9c2 cells were transfected with small interfering RNA (siRNA) using INTERFERin (Polyplus-transfection, NY, United States). The transfection effect was determined by Western blots. The sequence of siRNA1 was 5′-GCUACUGGGCAUCAUCAUA-3′; the sequence of siRNA2 was 5′-CCAGAAGACAGGCCAACAU-3′.

### Western Blot Assay

Protein was extracted from myocardium tissue or H9c2 cells. Here, 20 μg protein was separated by sodium dodecyl sulfate–polyacrylamide gel electrophoresis (SDS-PAGE) and transferred to 0.25-μm polyvinylidene difluoride (PVDF) membrane (Merck Millipore, Billerica, MA, United States). The membranes were incubated with primary antibodies overnight at 4°C. After being washed, the membranes were incubated with corresponding horseradish peroxidase (HRP)-coupled secondary antibodies. Blots were visualized by chemiluminescence reagents and were analyzed with ImageJ software.

### Co-immunoprecipitaion

The formation of necrosome and ubiquitination of RIP1 were assessed by immunoprecipitation method. H9c2 cells were lysed in NP-40 (Bosterbio, CA, United States). Then, protein was incubated with 2 μg RIP1 antibody or IgG overnight and then was added with 15 μl protein A/G agarose (Santa Cruz Biotechnology, TX, United States) for 2 h. After being washed, the beads were added 20 μl loading buffer and boiled. Supernatants were subjected to SDS-PAGE and analyzed.

### Immunohistochemical Staining

The sections were incubated with anti-4-HNE antibody overnight at 4°C (Abcam, ab46545, Cambridge, MA, United States) followed by being washed and stained with secondary antibodies. After that, 3,3′-diaminobenzidine (DAB) was used as chromogenic substrate. And the slices were counterstained with hematoxylin.

### Reverse Transcription Quantitative Polymerase Chain Reaction

To measure the mRNA levels of RIP1 in the myocardium tissues, RNA was extracted by EASYspin plus RNA extraction kit (Aidlab, Beijing, China) according to the instructions. Extracted RNA then was reverse transcribed to cDNA using Prime Script RT Master Mix (TaKaRa, Shiga, Japan). The amplifications and measurements were performed on ABI 7500 quantitative polymerase chain reaction instrument (Applied Biosystems; Thermo Fisher Scientific, MA, United States). The 2^–Δ^ ^Δ^ ^*CT*^ data of at least four independent experiments were recorded and analyzed.

### Statistical Analysis

Data were expressed as the means ± SEM and were analyzed by two-sided unpaired Student’s t-test. For multiple treatments, data were analyzed by one-way analysis of variance (ANOVA), followed by Tukey’s multiple comparisons test. *p* < 0.05 was considered statistical significance. All data were analyzed using GraphPad Prism version 5.0.

## Results

### Reperfusion Injury Induces Cell Necroptosis and Increases 4-Hydroxy-2-Nonenal Production in Mouse Hearts

**FIGURE 1:**
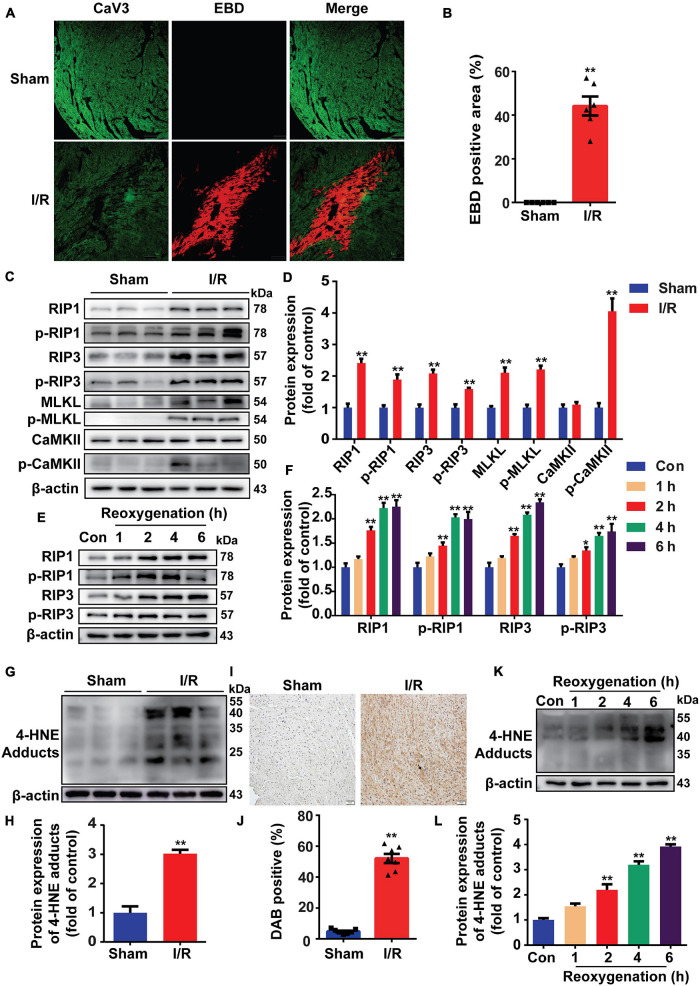
Reperfusion injury induces cell necroptosis and increases 4-hydroxy-2-non-enal (4-HNE) production in mouse hearts and H9c2 cells. **(A,B)** Representative photomicrographs and analysis of necrotic area (red) and viable cardiomyocytes (green) in the sections of mouse hearts (*n* = 6). Scale bar = 100 μm. **(C,D)** Representative Western blots and relative expression of receptor-interacting serine/threonine-protein kinase 1 (RIP1), p-RIP1, RIP3, p-RIP3, mixed lineage kinase domain-like pseudokinase (MLKL), p-MLKL, Ca^2+^/calmodulin-dependent protein kinase II (CaMKII), and p-CaMKII in mouse hearts (*n* = 6). **(E,F)** Representative immunoblots and relative expression of RIP1, p-RIP1, RIP3, and p-RIP3 in H9c2 cells under different reoxygenation times following hypoxia for 14 h (*n* = 5). **(G,H)** Protein expression and relative expression of 4-HNE adducts in mouse hearts (*n* = 6). **(I,J)** Representative immunohistochemical staining and analysis of mouse heart sections stained with anti-4-HNE antibodies (*n* = 7). Scale bar = 50 μm. **(K,L)** Protein expression of 4-HNE adducts in H9c2 cells under different reoxygenation times following hypoxia for 14 h (*n* = 5). *p < 0.05 vs. sham (control) group; **p < 0.01 vs. sham (control) group.

Mice were subjected to 30 min ischemia followed by 4 h reperfusion to induce MI/R injury. EBD was used to indicate necrosis area, while viable cardiomyocytes were labeled by CaV3. As shown in Figures 1A,B, MI/R injury obviously induced myocardial necrosis. During cell necroptosis, activation of RIP1 and RIP3 is essential, and both CaMKII and MLKL are considered executors of cell necroptosis (Zhe-Wei et al., 2018). To determine whether cell necroptosis occurred during MI/R injury, these proteins were detected, and we found that RIP1, p-RIP1, RIP3, p-RIP3, MLKL, p-MLKL, and p-CaMKII were all upregulated in reperfusion-injured hearts (Figures 1C,D). To confirm the effect of reperfusion injury on cell necroptosis, H9c2 cells were exposed to H/R stimulation. In line with the results *in vivo*, RIP1, RIP3, and their phosphorylation were all upregulated in H/R-treated cells (Figures 1E,F).

Since lipid peroxidation is markedly increased during MI/R injury, we then detected the change of 4-HNE and found that more 4-HNE was immunoblotted or immunochemically stained in MI/R-injured hearts (Figures 1G–J). Identically, 4-HNE was also increased in H/R-treated H9c2 cells (Figures 1K,L). Moreover, we detected 4-HNE *in vivo* and *in vitro* by Western blots and found that 4-HNE was not changed in ischemia (hypoxia) group (Supplementary Figures 1A,B).

### Detoxifying 4-Hydroxy-2-Nonenal Protects Against Myocardial Ischemia/Reperfusion Injury by Reducing Necroptosis

**FIGURE 2:**
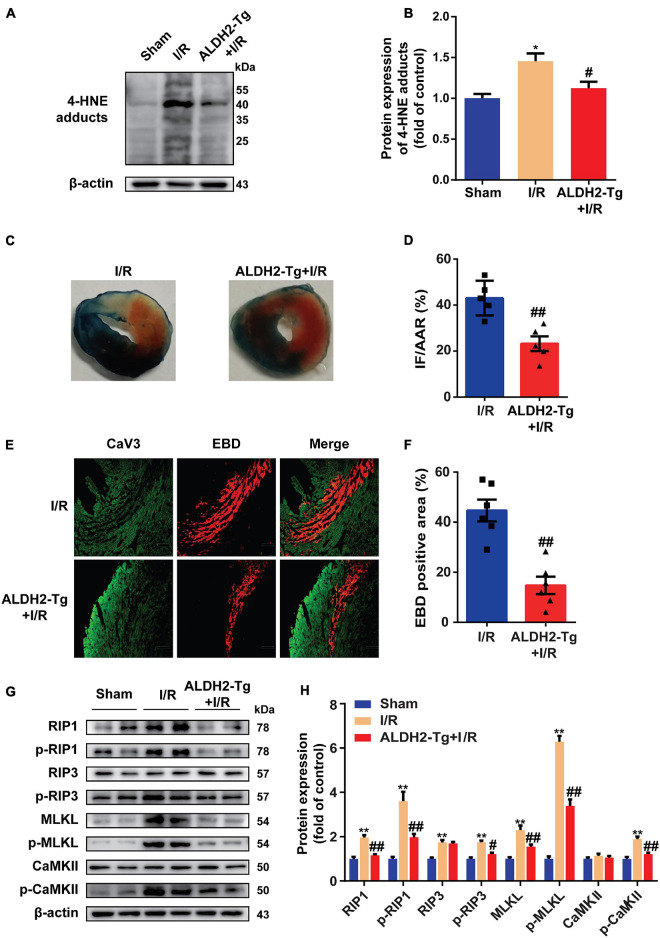
Detoxifying 4-hydroxy-2-non-enal protects against myocardial ischemia/reperfusion (MI/R) injury by reducing necroptosis. **(A,B)** Representative Western blots and relative expression of 4-HNE adducts in mouse hearts (*n* = 5). **(C,D)** Representative sections and statistical analysis of mouse hearts stained with Evans blue dye (EBD)/2,3,5-triphenyl tetrazolium chloride (TTC). White area stands for infarction area (IF); white and red area stands for area at risk (AAR) (*n* = 5). **(E,F)** Representative sections and analysis of heart sections stained with EBD and CaV3 (*n* = 6). Scale bar = 100 μm. **(G,H)** Representative immunoblots and relative expression of receptor-interacting serine/threonine-protein kinase 1 (RIP1), p-RIP1, RIP3, p-RIP3, mixed lineage kinase domain-like pseudokinase (MLKL), p-MLKL, Ca^2+^/CaMKII, and p-CaMKII in mouse hearts (*n* = 6). *p < 0.05 vs. sham group, **p < 0.01 vs. sham group; ^#^*p* < 0.05 vs. I/R group,^ ##^*p* < 0.01 vs. I/R group.

As ALDH2 is the main detoxifying enzyme of 4-HNE (Kimura et al., 2019), to elucidate the relationship between 4-HNE and necroptosis, ALDH2-Tg mice (Supplementary Figures 2A,B) and WT controls were exposed to MI/R injury. Less 4-HNE was detected in I/R-injured hearts from ALDH2-Tg group as compared with I/R group (Figures 2A,B). Cardiac function after I/R injury was improved by overexpression of ALDH2, as indicated by LVEF and LVFS (Supplementary Figure 2C). The myocardial infarct size was limited in ALDH2-Tg group compared to the I/R group (Figures 2C,D). Lactate dehydrogenase (LDH) was also decreased in ALDH2-Tg group (Supplementary Figure 2D). EBD-CaV3 staining showed that necrotic area was significantly reduced in ALDH2-Tg group (Figures 2E,F). Moreover, MI/R-induced upregulation of RIP1, p-RIP1, p-RIP3, MLKL, p-MLKL, and p-CaMKII was prevented by ALDH2 overexpression (Figures 2G,H). Because the formation of necrosome is critical to the happening of necroptosis (Chen et al., 2019), RIP1 was immunoprecipitated to detect the formation of necrosome and found that the binding of RIP1 to RIP3 was reduced in ALDH2-Tg group (Supplementary Figures 2E,F). These results demonstrated that 4-HNE might be a regulator of cell necroptosis in MI/R.

### 4-Hydroxy-2-Nonenal Induces Myocardial Necroptosis in Langendorff-Perfused Hearts

**FIGURE 3:**
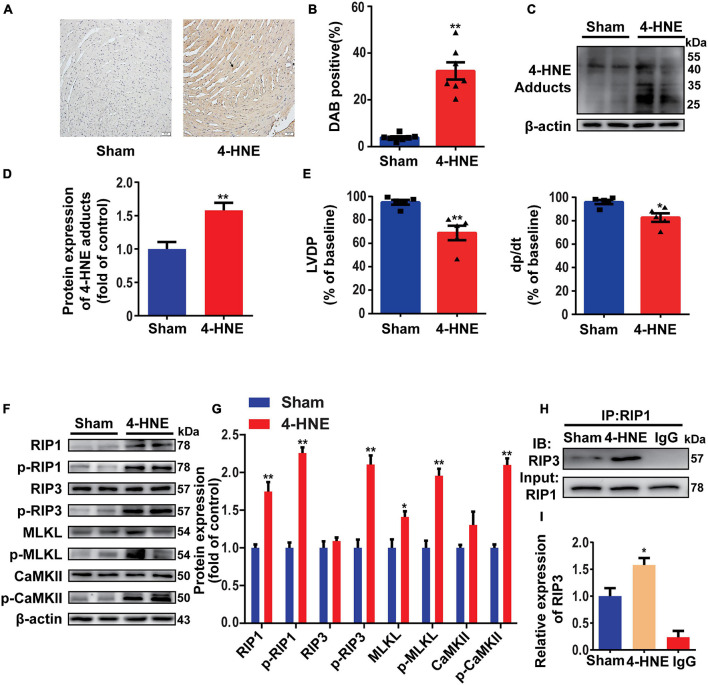
4-Hydroxy-2-non-enal induces myocardial necroptosis in Langendorff-perfused hearts. **(A,B)** Representative immunohistochemical staining and analysis of 4-HNE expression in mouse hearts (*n* = 7). Scale bar = 50 μm. **(C,D)** Representative Western blots and relative expression of 4-HNE adducts in mouse hearts (*n* = 6). **(E)** Cardiac function was evaluated by left ventricular developed pressure (LVDP) and left ventricular pressure rising rate (dp/dt) compared to the baseline (*n* = 5). **(F,G)** Representative Western blots and relative expression of receptor-interacting serine/threonine-protein kinase 1 (RIP1), p-RIP1, RIP3, p-RIP3, MLKL, p-MLKL, Ca^2+^/CaMKII, p-CaMKII in mouse hearts (*n* = 6). **(H,I)** Co-immunoprecipitation using anti-RIP1 antibody indicating the combination between RIP1 and RIP3 (*n* = 3). *p < 0.05 vs. sham group, **p < 0.01 vs. sham group.

To explore the role of 4-HNE in cell necroptosis, the Langendorff-perfused mouse heart model was used. After being stabilized for 20 min, the hearts were perfused with 60 μM 4-HNE or vehicle for 1 h. 4-HNE adducts were significantly increased in 4-HNE-perfused hearts, as indicated by both Western blot and immunohistochemical staining (Figures 3A–D). The cardiac function was impaired by 4-HNE perfusion, as indicated by the decreased LVDP and dp/dt compared to baseline (Figure 3E). Importantly, the perfusion of 4-HNE increased RIP1, p-RIP1, p-RIP3, MLKL, p-MLKL, and p-CaMKII (Figures 3F,G). Furthermore, more RIP3 was co-immunoprecipitated with RIP1 in 4-HNE-perfused hearts (Figures 3H,I). These results provided support for the crucial role of 4-HNE in cardiomyocyte necroptosis.

### 4-Hydroxy-2-Non-enal Induces Cell Necroptosis in H9c2 Cells

**FIGURE 4:**
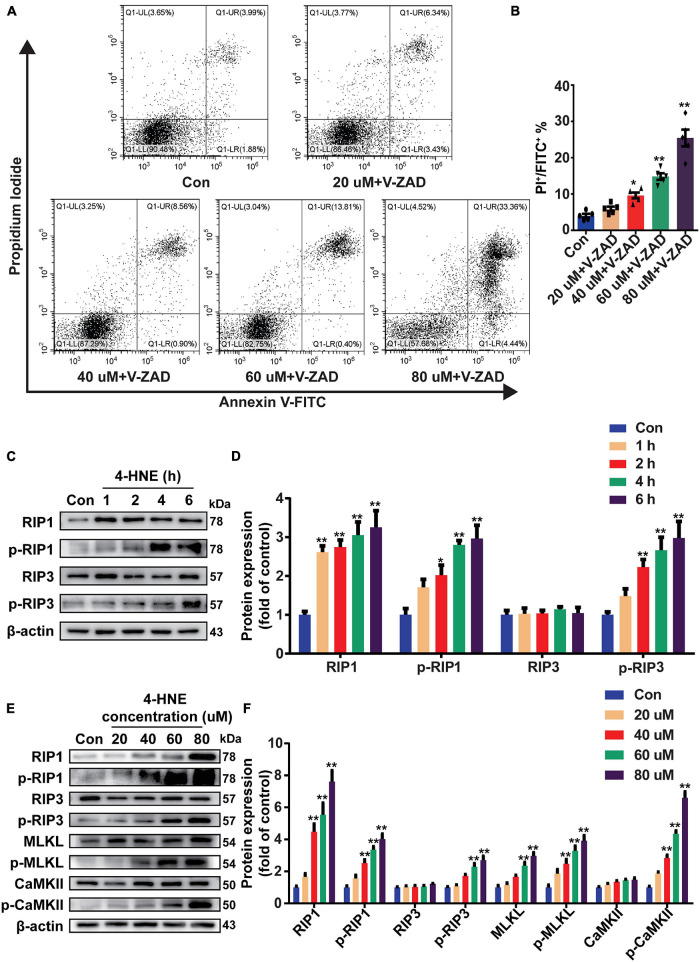
4-Hydroxy-2-non-enal induces cell necroptosis in H9c2 cells. **(A,B)** Necroptotic cells were determined using flow cytometry stained with Annexin V and propidium iodide (PI), and the cell necroptosis ratio was demonstrated as the percentage of Annexin V^+^/PI^+^ cells (*n* = 5). **(C,D)** Representative Western blots and relative expression of RIP1, p-RIP1, RIP3, and p-RIP3 under time gradients of 4-HNE treatment in H9c2 cells (*n* = 5). **(E,F)** Representative immunoblots and relative expression of RIP1, p-RIP1, RIP3, p-RIP3, MLKL, p-MLKL, Ca^2+^/CaMKII, and p-CaMKII in H9c2 cells treated with different concentrations of 4-HNE (*n* = 5). *p < 0.05 vs. control group, **p < 0.01 vs. control group.

To confirm the effect of 4-HNE on necroptosis, H9c2 cells were pretreated with Z-VAD-FMK (the pan-caspase inhibitor and was used to inhibit apoptosis) and then were stimulated with 4-HNE. Necroptotic cells were measured using flow cytometry and were defined as Annexin V^+^/PI^+^ cells. 4-HNE promoted cell necroptosis (Figures 4A,B). Moreover, RIP1, p-RIP1, and p-RIP3 were all upregulated by 4-HNE in a time-dependent manner (Figures 4C,D). Meanwhile, RIP1, p-RIP1, p-RIP3, MLKL, p-MLKL, and p-CaMKII were also upregulated by 4-HNE in a dose-dependent manner (Figures 4E,F).

### Receptor-Interacting Serine/Threonine-Protein Kinase 1 Mediates the Effect of 4-Hydroxy-2-Nonenal on Cardiomyocyte Necroptosis

**FIGURE 5:**
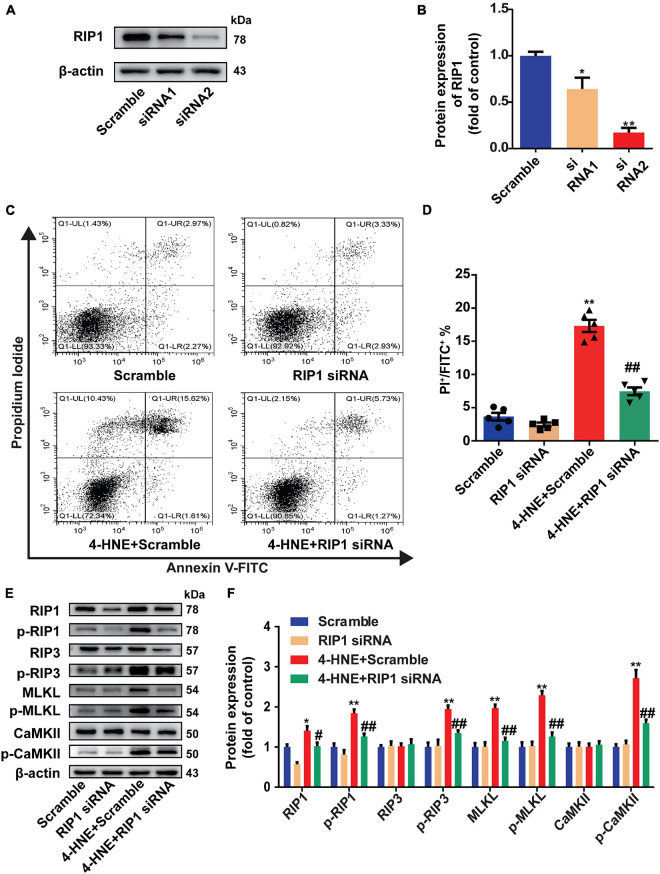
Receptor-interacting serine/threonine-protein kinase 1 mediates the effect of 4-HNE on cardiomyocyte necroptosis. **(A,B)** Representative Western blots and relative expression of RIP1 in H9c2 cells treated with scrambled or RIP1-targeted siRNAs (*n* = 3). **(C,D)** Necroptotic cells were analyzed by Annexin V/PI staining using flow cytometry (*n* = 5). The necroptotic cells were indicated as Annexin V^+^/PI^+^ cells. **(E,F)** Representative immunoblots and relative expression of RIP1, p-RIP1, RIP3, p-RIP3, MLKL, p-MLKL, Ca^2+^/CaMKII, and p-CaMKII in different groups treated with scramble or RIP1 siRNA with or without 4-HNE stimulation in H9c2 cells (*n* = 5). *p < 0.05 vs. scramble group, **p < 0.01 vs. scramble group; ^#^*p* < 0.05 vs. 4-HNE + scramble group, ^##^*p* < 0.01 vs. 4-HNE + scramble group.

Receptor-interacting serine/threonine-protein kinase 1 is necessary for the regulation of RIP3 and the downstream pathways (Liu et al., 2019). To determine the critical role of RIP1 in the regulation of cardiomyocyte necroptosis by 4-HNE, the H9c2 cells were transfected with scramble or RIP1 siRNA (Figures 5A,B). The effect of 4-HNE (60 μM) on cell necroptosis was mitigated by knockdown of RIP1 (Figures 5C,D). Moreover, the 4-HNE-induced changes of p-RIP1, p-RIP3, MLKL, p-MLKL, and p-CaMKII were prevented by RIP1 deficiency (Figures 5E,F).

### 4-Hydroxy-2-Nonenal Reduces the Ubiquitin-Dependent Degradation of Receptor-Interacting Serine/Threonine-Protein Kinase 1

**FIGURE 6:**
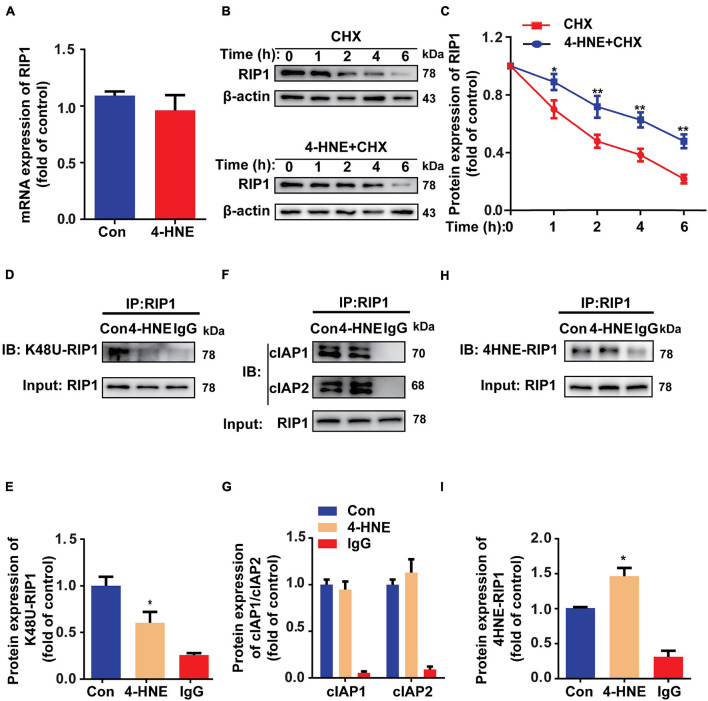
4-Hydroxy-2-non-enal reduces the ubiquitin-dependent degradation of RIP1. **(A)** RIP1 mRNA expression in H9c2 tested by quantitative real-time polymerase chain reaction (*n* = 5). **(B,C)** Protein expression of RIP1 in H9c2 cells treated with cycloheximide (CHX) and 4-HNE detected by Western blot (*n* = 5). **(D,E)** Co-immunoprecipitation using anti-RIP1 antibody showed the K48 ubiquitination of RIP1 (*n* = 3). **(F,G)** Co-immunoprecipitation using anti-RIP1 antibody to detect combination between RIP1 and cIAP1 or cIAP2 (*n* = 3). **(H,I)** Co-immunoprecipitation using anti-RIP1 antibody to detect the combination between RIP1 and 4-HNE (*n* = 3). *p < 0.05 vs. control group, **p < 0.01 vs. control group.

To investigate how 4-HNE upregulates the protein level of RIP1, the protein expression and degradation of RIP1 were both examined. The mRNA level of RIP1 was not changed by 4-HNE stimulation (Figure 6A). To assess the protein degradation of RIP1, CHX was used to inhibit the gene transcription, and we found that less protein was degraded in 4-HNE group (Figures 6B,C). These results suggested that 4-HNE upregulated RIP1 *via* preventing the protein degradation. K-48-linked polyubiquitination is a common type of ubiquitination that is associated with the degradation of RIP1 by proteasome (Samant et al., 2018). To verify whether 4-HNE regulated RIP1 degradation by decreasing the ubiquitination of RIP1, the K-48-linked polyubiquitination of RIP1 was detected, and we found that less K-48 ubiquitin was co-immunoprecipitated with RIP1 after 4-HNE stimulation (Figures 6D,E). Furthermore, the process of protein ubiquitination is divided into the combination of substrate protein with ubiquitin ligases and the transfer of ubiquitin to the substrate protein (Komander, 2009). To figure out which process was regulated by 4-HNE, cIAP1, and cIAP2, the important direct E3 ubiquitin ligases of RIP1 (Wang et al., 2008), were coimmunoprecipitated with RIP1, and we found the binding of cIAP1 and cIAP2 to RIP1 was not changed by 4-HNE stimulation (Figures 6F,G). To further underlie the mechanism of reduced ubiquitination of RIP1 by 4-HNE, the carbonylation of RIP1 was examined, and more 4-HNE was co-immunoprecipitated with RIP1 (Figures 6H,I). Taken together, we speculate that 4-HNE might prevent the K48 polyubiquitination-dependent protein degradation of RIP1 by enhancing the carbonylation of RIP1.

## Discussion

**FIGURE 7:**
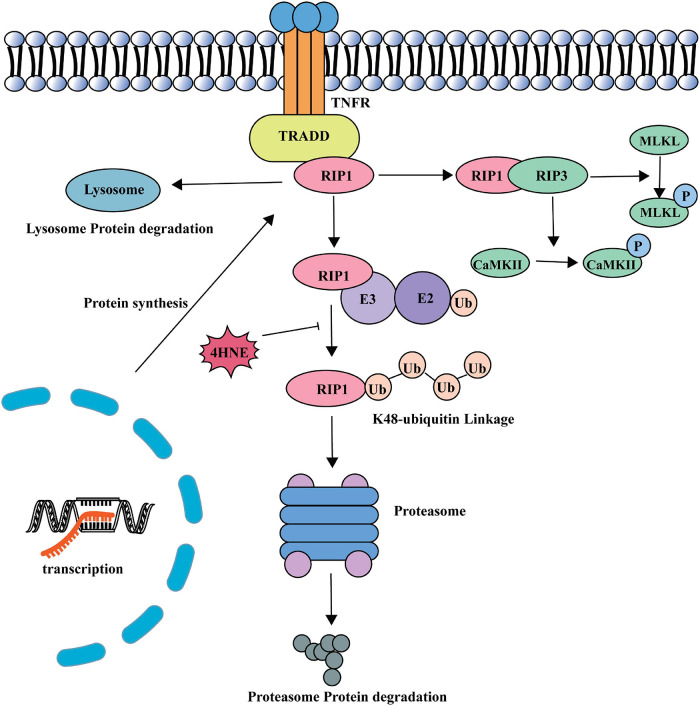
A model of the role of 4-HNE in regulating cardiomyocyte necroptosis during MI/R.

In this study, we found that 4-HNE played an important role in myocardial necroptosis during MI/R injury. 4-HNE induced cardiomyocyte necroptosis by increasing RIP1 and RIP3 and the phosphorylation of the two proteins. Subsequently, the increase of MLKL and the phosphorylation of MLKL and CaMKII also evidenced that 4-HNE promoted cell necroptosis. Further studies found that 4-HNE favored the stabilization of RIP1 by suppressing K48 polyubiquitination of RIP1, leading to less protein degradation (Figure 7).

Myocardial ischemia reperfusion injury is characterized as the large burst of cardiomyocyte deaths (Whelan et al., 2010). Increasing evidence shows that necroptosis is the major type of programmed cell necrosis during MI/R. Preventing necroptosis was able to reduce myocardial infarct zone by nearly one third in animal MI/R models (Dmitriev et al., 2013; Zhang et al., 2014; Koudstaal et al., 2015).

4-Hydroxy-2-nonenal, an important product during oxidative stress, is abundantly produced during MI/R injury (Mali and Palaniyandi, 2014). 4-HNE regulates pathological processes through various ways. 4-HNE can bind to cysteine, histidine, and lysine amino residues by Michael reactions (Dubinina and Dadali, 2010). 4-HNE can also directly modify four DNA bases and then affect diverse biological processes (Zhong and Yin, 2015). Previous studies proved that 4-HNE regulated cell apoptosis or autophagy in different ways (Dodson et al., 2017; Yang et al., 2019). However, whether 4-HNE contributes to cell necroptosis was never reported. In the present study, we found that 4-HNE played an important role in cardiomyocyte necroptosis during MI/R injury or in the isolated perfused heart.

Necroptosis is usually initiated by the combination between pro-inflammatory factors and their corresponding receptors. The most well-studied pathway in cardiovascular diseases is tumor necrosis factor receptor 1 (TNFR1) signaling (Zhu and Sun, 2018). Once it is activated, RIP1, TNFR-associated death domain (TRADD), TNFR-associated factor (TRAF), and other factors are recruited to the intracellular part of TNFR1 to form necroptosis complex I. After the TNFR1 endocytosis, complex I shifts into complex II, which recruits and activates RIP3. MLKL and CaMKII are regarded as executors of necroptosis and activated by RIP3 (Galluzzi et al., 2017).

Receptor-interacting serine/threonine-protein kinase 1 has emerged as a vital upstream kinase that controls multiple cellular pathways involved in regulating cell death (Christofferson et al., 2014). RIP1 induces necroptosis by recruiting RIP3 and forming necrosome (Li et al., 2012). In this study, we found that increased RIP1 played a critical role in 4-HNE-induced cardiomyocyte necroptosis. RIP1 kinase is intricately regulated by ubiquitination, deubiquitination, and phosphorylation (Christofferson et al., 2014). Ubiquitination is a kind of post-translation modification during which ubiquitin protein is attached to substrate proteins. RIP1 can recruit E3 ligases such as cIAP1 and cIAP2, which mediate K11, K48, K63, and linear polyubiquitination of RIP1 (Witt and Vucic, 2017). K48-linked ubiquitin modifications of RIP1 favor the combination with proteasomes and result in degradation of RIP1, while K63-linked ubiquitin modifications of RIP1 promote the activation of nuclear factor (NF)-κB (Lu et al., 2013; Ofengeim and Yuan, 2013). In this study, we found that 4-HNE reduced the K48 polyubiquitination of RIP1. Additionally, 4-HNE mainly affects the conjugation of ubiquitin molecules to the RIP1 instead of engagement of E3 ligases, as indicated by Figures 6F,G. As shown in Supplementary Figure 3, the structure of RIP1 is composed of lysine, cysteine, and histidine; thus, it is possible that 4-HNE modifies RIP1 directly (Ofengeim and Yuan, 2013). Thus, we speculate that the carbonylation of RIP1 might affect the ubiquitin binding sites and limit the binding of K48-linked ubiquitin.

In summary, we demonstrated that elevated 4-HNE directly combined with RIP1, which contributed to the decreasing of ubiquitin-mediated RIP1 degradation, thus resulting in activating the necroptosis pathway and promoting the myocardium necroptosis.

## Data Availability Statement

The raw data supporting the conclusion of this article will be made available by the authors, without undue reservation.

## Ethics Statement

The animal study was reviewed and approved by Animal Ethics Committee of Qilu Hospital of Shandong University.

## Author Contributions

YC conceived the project. SW and JW planned and designed the experiments. XZ performed most of the experimental work and analyzed the data. WW performed some experimental work, interpreted the results, and drafted the manuscript. SS and JL participated in the performing of mouse I/R surgery. SC and RL participated in maintenance of H9c2 cell. TX and QY planned the Langendorff model and finished it. SW wrote the final version of the article. All authors contributed to the article and approved the submitted version.

## Conflict of Interest

The authors declare that the research was conducted in the absence of any commercial or financial relationships that could be construed as a potential conflict of interest.

## Publisher’s Note

All claims expressed in this article are solely those of the authors and do not necessarily represent those of their affiliated organizations, or those of the publisher, the editors and the reviewers. Any product that may be evaluated in this article, or claim that may be made by its manufacturer, is not guaranteed or endorsed by the publisher.
