# Supplementary material for: 4-Hydroxy-2-Nonenal Promotes Cardiomyocyte Necroptosis via Stabilizing Receptor-Interacting Serine/Threonine-Protein Kinase 1
Source: Front Cell Dev Biol. 2021 Oct 1;9:721795. doi: 10.3389/fcell.2021.721795 (PMC8517475; doi:10.3389/fcell.2021.721795)
Supplement: Supplementary file 1 [file Data_Sheet_1.docx]

**Supplemental Data**


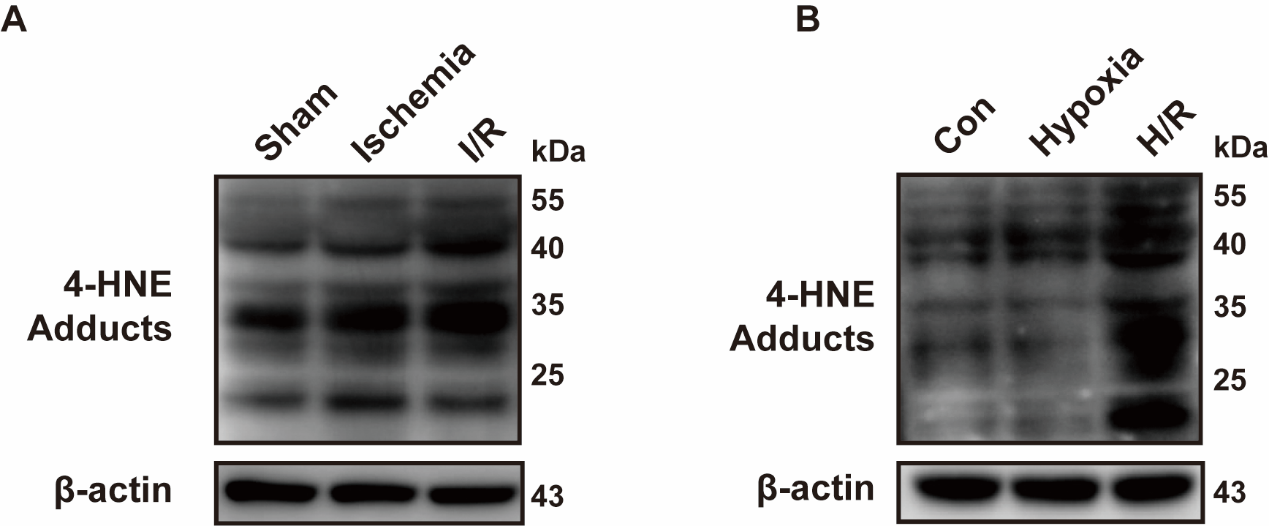


**Supplementary Figure 1.** The expression of 4-HNE-adducts under ischemia (hypoxia) condition. **(A)** Protein expression of 4-HNE in mouse hearts. (ischemia for 30 min, n=5). **(B)** Protein expression of 4-HNE in H9c2 cells. (hypoxia for 12 h, n=5).


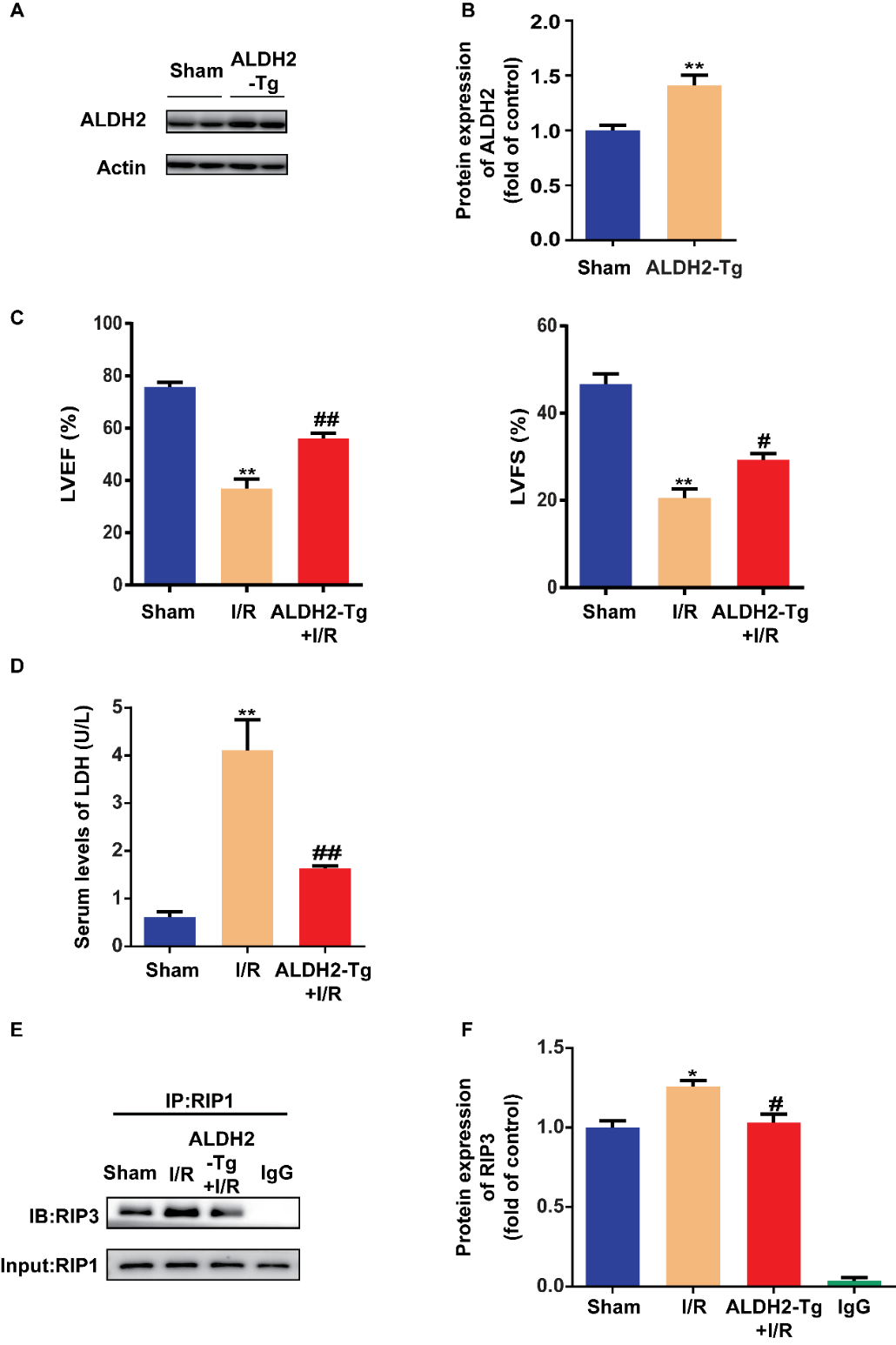


**Supplementary Figure 2.** Overexpression of ALDH2 decrease the levels of 4-HNE adducts and protect the hearts for I/R injury. **(A, B)** Representative western blots and analysis of ALDH2 in Sham group and ALDH2-Tg group. (n=5). **(C)** Cardiac function was evaluated by echocardiography and indicated by left ventricular ejection function (LVEF) and left ventricular fraction shortening (LVFS). (n=6). **(D)** Serum levels of LDH were detected by ELISA. (n=4). **(E, F)** Co-immunoprecipitation using RIP1 antibody to detect the combination between RIP1 and RIP3. (n=3). Data are presented as mean ± SEM. **P* < .05 vs Sham group, ***P* < .01 vs Sham group.


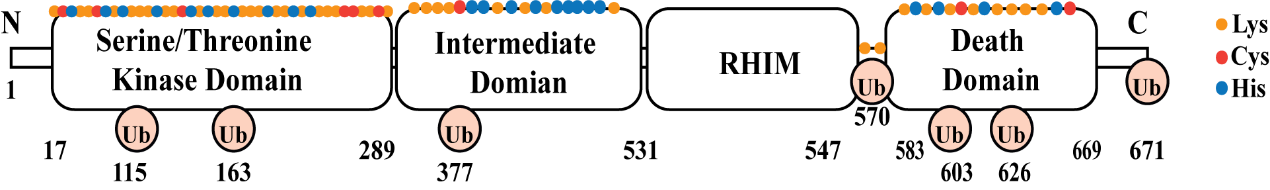


**Supplementary Figure 3.** The domain structure of RIP1 and distribution of lysine, cysteine, histone, ubiquitin sites on the RIP1.
